# Once Upon a Time: Storytelling as a Tool to Reduce Anxiety and Foster Emotional Expression in Schoolchildren

**DOI:** 10.1192/j.eurpsy.2026.11131

**Published:** 2026-07-31

**Authors:** S. Pérez-Sánchez, C. Pérez-Sánchez, E. Gonzalez-Reigadas, I. Martín-Herrero

**Affiliations:** 1 Psychiatry; 2 Medicina Familia, Servicio Murciano de Salud, Murcia, Spain

## Abstract

**Introduction:**

Anxiety disorders are the most frequently diagnosed psychological problems in children. If left untreated, their natural course may lead to significant negative repercussions on academic performance, social relationships, and family functioning. Group storytelling activities in the classroom allow children to be exposed to emotions and to identify with peers, thereby facilitating the internalization and expression of these emotions.

**Objectives:**

1. To determine the frequency and characteristics of anxiety symptoms in a community-based school population.
2. To evaluate whether group therapy techniques based on storytelling improve the expression of emotions in children.

**Methods:**

A prospective study was conducted over 9 months in a primary school. The intervention consisted of four monthly classroom sessions combining storytelling and group reflection. The SCAST scale was administered before and after the intervention. Data were analyzed using SPSS v23.0.

**Results:**

A total of 110 primary school students participated (37.7% boys, 62.3% girls). By age, 41.7% were 7 years old and 56.3% were 8 years old. At baseline, 25.2% of participants presented high anxiety scores, most frequently among girls (66%). The most prevalent symptoms were separation anxiety and physical fears (2.3%) and social phobia (1.7%). Following the intervention, a significant reduction in the mean global score was observed (mean decrease: 21.2; SD = 9.8). A greater reduction in global scores was noted among girls, although the difference did not reach statistical significance.

**Conclusions:**

Exploring childhood fears and anxieties within the school setting is essential to provide children with tools for emotional regulation. Storytelling appears to be an effective and feasible intervention, easily integrated into the classroom context, that promotes emotional expression and supports both academic and personal development. Group therapy techniques based on storytelling may therefore represent a valuable strategy to improve emotional well-being in children.

**Disclosure of Interest:**

None Declared

